# Higher Matrix Stiffness Upregulates Osteopontin Expression in Hepatocellular Carcinoma Cells Mediated by Integrin β1/GSK3β/β-Catenin Signaling Pathway

**DOI:** 10.1371/journal.pone.0134243

**Published:** 2015-08-17

**Authors:** Yang You, Qiongdan Zheng, Yinying Dong, Yaohui Wang, Lan Zhang, Tongchun Xue, Xiaoying Xie, Chao Hu, Zhiming Wang, Rongxin Chen, Yanhong Wang, Jiefeng Cui, Zhenggang Ren

**Affiliations:** 1 Liver Cancer Institute, Zhongshan Hospital, Fudan University & Key Laboratory of Carcinogenesis and Cancer Invasion, Ministry of Education, 136 Xue Yuan Road, Shanghai, 200032, PR China; 2 Department of Interventional Radiology, Shanghai Cancer Center, Fudan University, Shanghai, 200032, PR China; 3 Department of Urology, Zhongshan Hospital, Fudan University, Shanghai, 200032, PR China; 4 Department of Oncology, Zhongshan Hospital Subdivision, Fudan University, Shanghai, 200052, PR China; The University of Hong Kong, HONG KONG

## Abstract

Increased stromal stiffness is associated with hepatocellular carcinoma (HCC) development and progression. However, the molecular mechanism by which matrix stiffness stimuli modulate HCC progress is largely unknown. In this study, we explored whether matrix stiffness-mediated effects on osteopontin (OPN) expression occur in HCC cells. We used a previously reported in vitro culture system with tunable matrix stiffness and found that OPN expression was remarkably upregulated in HCC cells with increasing matrix stiffness. Furthermore, the phosphorylation level of GSK3β and the expression of nuclear β-catenin were also elevated, indicating that GSK3β/β-catenin pathway might be involved in OPN regulation. Knock-down analysis of integrin β1 showed that OPN expression and p-GSK3β level were downregulated in HCC cells grown on high stiffness substrate compared with controls. Simultaneously, inhibition of GSK-3β led to accumulation of β-catenin in the cytoplasm and its enhanced nuclear translocation, further triggered the rescue of OPN expression, suggesting that the integrin β1/GSK-3β/β-catenin pathway is specifically activated for matrix stiffness-mediated OPN upregulation in HCC cells. Tissue microarray analysis confirmed that OPN expression was positively correlated with the expression of LOX and COL1. Taken together, high matrix stiffness upregulated OPN expression in HCC cells via the integrin β1/GSK-3β/β-catenin signaling pathway. It highlights a new insight into a pathway involving physical mechanical signal and biochemical signal molecules which contributes to OPN expression in HCC cells.

## Introduction

Osteopontin (OPN), also known as secreted phosphoprotein 1 (SPP1), is involved in a series of physiological and pathological processes including cell attachment, migration, invasion, proliferation, tissue remodeling, bone formation, and inflammation [1]. In recent years, increasing evidence has defined the value of OPN as a candidate biomarker and drug target for many types of cancers, such as hepatocellular carcinoma (HCC) [2], breast cancer [3], ovarian cancer [4], cervical cancer [5], and gastric cancer [5]. Notably, high expression of OPN in HCC tissue, detectable both at transcriptional and translational levels, as well as high level of serum OPN, are strongly predictors of poor prognosis and diagnosis of HCC [6, 7]. Several signaling pathways, such as PI3K/AKT, MAPK, Wnt/β-catenin, and NFκβ signaling pathways, modulate the activation of OPN expression [8–10]. However, all of these pathways are activated by biochemical signal stimuli, not physical signal stimuli.

HCC is the fifth common cancer and the third leading cause of cancer-related mortality worldwide [11]. Approximately 30% of the patients with cirrhosis develop HCC, and 90% of HCC patients have a history of cirrhosis or advanced fibrosis [12]. Increased stromal stiffness precedes and accompanies fibrosis in chronic liver diseases [13, 14], and higher liver stiffness, measured by transient elastography, enhances the risk of HCC occurrence in patients with chronic hepatitis C and facilitates its progress. At present, liver matrix stiffness is considered as a strong predictor in clinic for HCC diagnosis and prognosis [15]. The physical features of the adjacent environment of tumor cells, particularly matrix topology and stiffness, influence cancer initiation and progression, which is a topic of great interest to oncologists. However, the molecular mechanism by which matrix stiffness stimuli modulate HCC progress remains largely unknown. In general, the size of biopolymer fibers and the density of the fiber network determine matrix stiffness level [16]. Matrix rigidity influences the growth, viability, differentiation, and motility of cells [17–19]. Several studies demonstrate that matrix stiffness contributes to the proliferation, development, and chemoresistance of HCC through FAK, Erk, Pkb/Akt, and STAT3 pathways [20, 21] and upregulates VEGF expression via activation of the integrin β1/PI3K/Akt pathway [22]. Lysyl oxidase-like 2 (LOXL2) can promote intrahepatic metastasis of HCC by increasing tissue stiffness [23]. Integrin β1 is the key protein receptor bridging matrix stiffness and intercellular signals [24]. Expression of integrin β1 is regulated by the mechanical stiffness of the ECM and correlates with the invasion and metastasis of HCC in patients with cirrhosis [25]. Integrin can regulate a serial of pathways such as FAK/AKT[25], Ras/ Raf, MEK as well as MEKKs (MAPK/ ERK Kinase Kinase), PAK (p21-Activated Kinase), MEKs (MAPK /ERK Kinases), Vav, and JNK (c-Jun NH2-terminal kinase)[26]. In ovarian carcinoma, multivalent integrin engagement results in increased internalization of E-cadherin, inhibition of GSK-3β, elevated levels of nuclear β-catenin, increased β-catenin-regulated promoter activation, and transcriptional activation of Wnt/β-catenin target genes[27]. On the other hand, the dephosphorylation activates GSK3β, leading to degradation of β-catenin and subsequent loss of TCF/LEF (T cell factor1/lymphoid enhancer factor1) activity[28]. Additionally, Tcf-4 enhanced cell invasion in breast cancer cells via transcriptional enhancement of OPN expression[29]. However, the relationship between OPN expression and matrix rigidity, especially whether matrix stiffness activates OPN expression and whether integrin/β-catenin pathway is involved in this process remain poorly understood. In the present study, using an in vitro culture system with tunable stiffness, we explore the underlying molecular mechanism of matrix stiffness-mediated effects on OPN expression in HCC cells and highlight a new insight into a pathway involving physical mechanical signal and biochemical signal molecules which contributes to OPN upregulation.

## Materials and Methods

### In vitro system of mechanically tunable COL1-coated polyacrylamide gel

An in vitro system of mechanically tunable COL1-coated polyacrylamide gel was established as previously described [22]. Briefly, polyacrylamide gels with different mechanical stiffness levels were prepared by mixing 10% acrylamide and 0.01% to 0.5% bis-acrylamide in a HEPES- buffered solution (pH 8) supplemented with 10% ammonium persulfate (APS, 1/100 volume) and TEMED (1/100 volume), and then the formed gels were further crosslinked and coated with 0.1 mg/ml COL-1 solution (BD) suitable for cell culture.

### HCC cells and cell culture

Huh7 cells (ATCC, USA) were cultured in Dulbecco’s modified Eagle’s medium (Gibco, USA) supplemented with 10% fetal bovine serum (FBS; Biowest, South America Origin) and 1% penicillin/streptomycin (Gibco, USA). Hep3B cells (ATCC, USA) were cultured in minimum essential medium (Gibco, USA) supplemented with 10% FBS and 1% penicillin/streptomycin. Approximately 3×10^5^ HCC cells in 0.3 ml of medium were seeded onto a thin layer of COL1-coated polyacrylamide gel with tunable stiffness for 2 h at room temperature. Subsequently, 6 ml of culture medium was added to the dish, and the cells were further incubated at 37°C for 24 h or 48h.

### Western blot and Real-time PCR Assays

Please see the detailed procedures of Western blot and **real-time PCR assays** in S1 File.

### Enzyme-linked immunosorbent assay (ELISA)

Concentration of OPN in culture supernatant was measured by ELISA following the manufacturer’s instructions (Boyan Biosciences Company, Shanghai, China.)

### Stable knock-down expression of integrin β1 in HCC with lentiviral vectors

Small interfering RNAs (siRNAs) targeting the human integrin β1 gene were designed by the Shanghai GeneChem, Co. Ltd, China. The optimal sequence of siRNA against human integrin β1 (5′-CCTCCAGATGACATAGAAA-3′) was then cloned into the plasmid GV112. Lentivirus preparations were produced by Shanghai GeneChem, Co. Ltd, China. The resulting shRNA human integrin β1 sequence was confirmed by PCR and sequencing analysis. Different siRNAs were screened by cotransfection with a human integrin β1 cDNA plasmid into HEK293T cells with Lipofectamine 2000 (Invitrogen Corporation, Carlsbad, CA, USA). The viral supernatant was harvested 48 h after transfection, and the viral titer was determined. The viral supernatant was added into the target HCC cells (at multiplicity of infection = 10) with ENi.S and 5 μg/ml polybrene to obtain stably-transfected HCC cells with integrin β1 knock-down.

### GSK-3β inhibition assay

GSK-3β inhibitor (Selleck, China) was diluted into the final concentration of 3 μM [30] with complete culture medium. HCC cells cultured on high stiffness substrate were treated with GSK-3β inhibitor for 48 h and then were collected for Western blot analysis.

### Tissue microarray and immunohistochemistry

Tissue microarray slide was constructed as previously described in our work [22]. In brief, based on the results of hematoxylin and eosin-stained tumor tissue slides, two cores containing optimal tumor content were positioned and obtained by punch cores from a formalin-fixed, paraffin-embedded tumor tissue. The immunohistochemistry procedure is described in S1 File

### Expression levels of MMP9 gene under exogenous OPN intervention in HCC cells grown on different stiffness substrates

Please see the detailed procedures in S1 File.

### Statistical analysis

Statistical analysis of values for comparison between two groups was performed using two-tailed Student’s t test. One-way ANOVA and multiple linear regression were used to analyze the correlation among the expression of LOX, COL, and OPN. Data were expressed as mean ± SD, and p < 0.05 was considered statistically significant.

## Results

### Higher matrix stiffness upregulates OPN expression in HCC cells and activates the GSK3β/β-catenin signaling pathway in vitro

**Fig 1:**
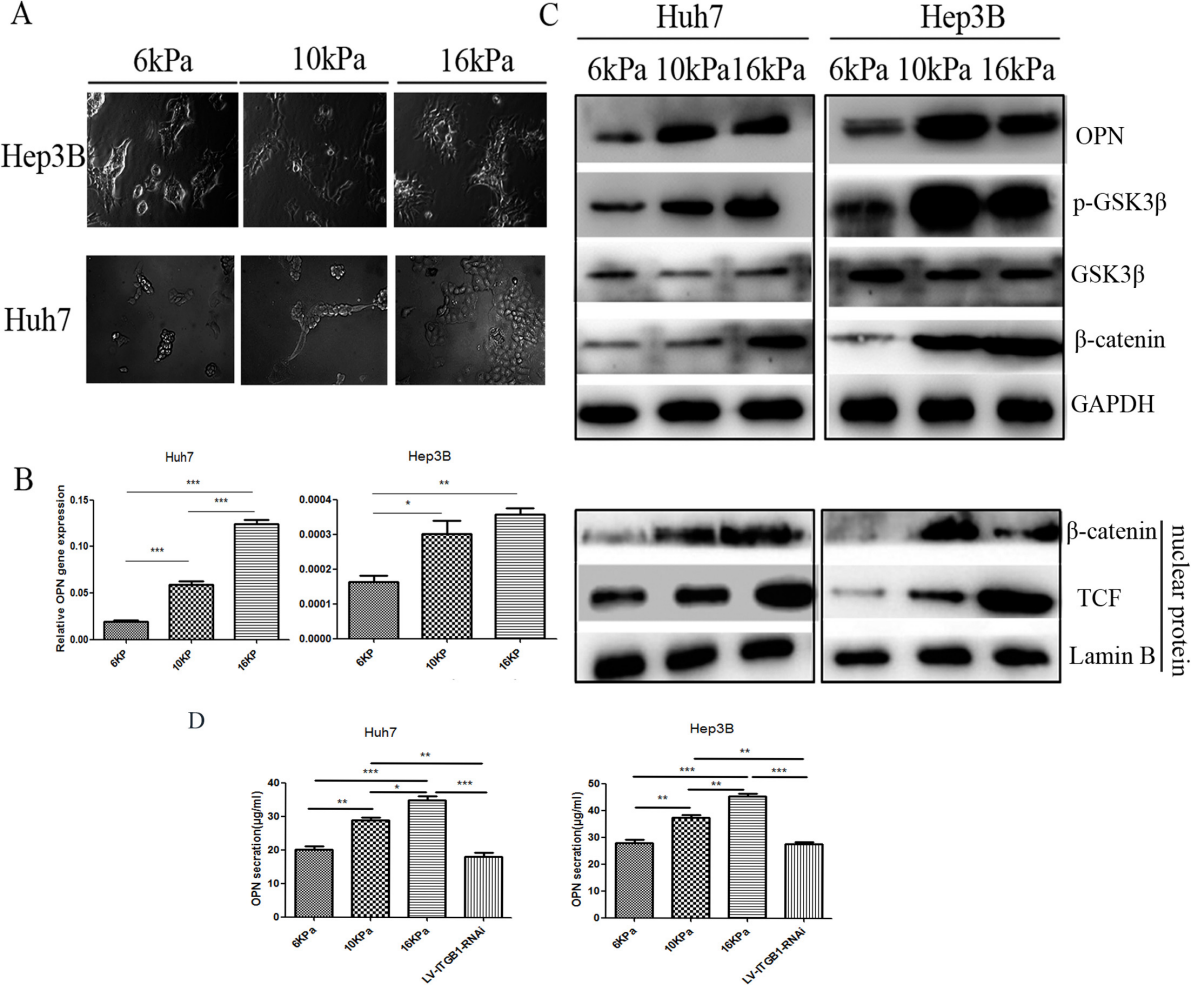
Higher matrix stiffness upregulates OPN expression in HCC cells and activates the GSK3β/β-catenin signaling pathway. (A) Morphology of Hep3B and Huh7 cells cultured on low stiffness substrate (6 kPa), medium-stiffness substrate (10 kPa) and high-stiffness substrate (16 kPa). (B) mRNA expression of OPN in Hep3B and Huh7 cells cultured on different stiffness substrates. (C) Increasing matrix stiffness upregulates OPN expression and activates the GSK3β/β-catenin signaling pathway in both Hep3B and Huh7 cells. (D) Osteopontin concentrations in supernatant of Huh7 and Hep3B cells cultured on different stiffness substrates and HCC cells with LV-INTGB1-RNAi cultured on high stiffness substrate. In each case, error bars represent SD, *p < 0.05, **p < 0.01, ***p<0.0001.

High-stiffness substrate (16 kPa), medium-stiffness substrate (10 kPa), and low-stiffness substrate (6 kPa) were used to represent the stiffness level of different stages of cirrhosis, fibrosis, and normal liver tissue, respectively. Huh7 and Hep3B cells were cultured on these substrates to investigate the effect of matrix stiffness on OPN expression. Fig 1A shows that Huh 7 and Hep3B cells grew well on the cell culture platform, and their morphologies altered from round to fully spread under different stiffness conditions. Moreover, the transcriptional (Fig 1B) and translational (Fig 1C) levels of OPN were significantly upregulated in both Huh7 and Hep3B cells with increasing matrix stiffness. Simultaneously, the phosphorylation level of GSK3β and the expression of nuclear β-catenin were also upregulated in two HCC cells (Fig 1C). Additionally, the concentration of OPN in supernatant of HCC cells was higher on higher stiffness substrate than that on low stiffness substrate. Moreover, the level of the secreted OPN in supernatant of HCC cells with integrin β1 knock-down cultured on 16kPa stiffness substrate had also decreased (Fig 1D). Subsequently, we added recombinant human OPN into culture medium of HCC cells grown on different stiffness substrates to measure the expression of invasion relative gene MMP9. We found that Huh7 and Hep3B cells in OPN interventional groups highly expressed MMP9 as compared with that of the control HCC cells, indicating invasion ability of HCC cells is enhanced under OPN stimulation (S1 Fig). These results suggest that increasing matrix stiffness enhanced OPN expression and this upregulation may be associated with activation of GSK-3β/β-catenin/TCF. The Wnt/β-catenin pathway has been well documented to involve in OPN regulation [10]. Based on our results, we propose that higher matrix stiffness may trigger a Wnt-independent β-catenin pathway to modulate the OPN expression in HCC cells.

### Higher matrix stiffness activates GSK3β/β-catenin signaling pathway mediated by integrin-β1 to modulate OPN expression

**Fig 2:**
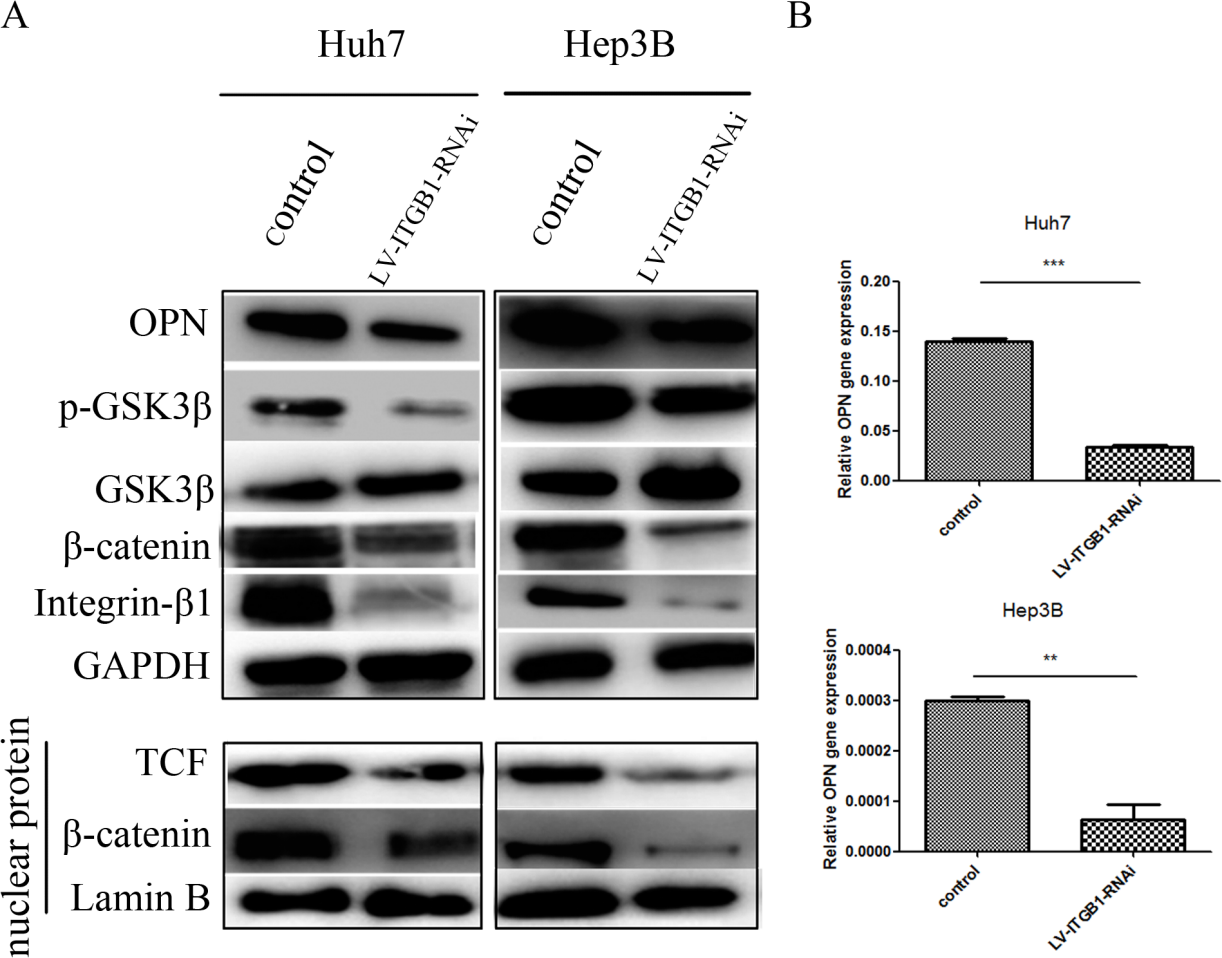
Higher matrix stiffness activates GSK3β/β-catenin signaling pathway mediated by integrin-β1 to modulate OPN expression. (A) Levels of P-GSK3β and β-catenin were attenuated in HCC cells infected with LV-INTGB1-RNAi on high stiffness substrate. Nuclear expression of β-catenin and TCF dropped sharply, and the expression of OPN also decreased significantly. (B) OPN expression Huh7 and Hep3B cells infected with LV-ITGB1-RNAi on high stiffness substrate. In each case, error bars represent SD, *p < 0.05, **p < 0.01, ***p<0.0001.

Integrin functions mainly in delivering matrix stiffness signals into cell and initiating a cascade of downstream events to influence cell function or biological behaviors. Among all the integrin subtypes, integrin β1 as a leading subtype that has been reported to be differentially expressed in HCC cells between higher stiffness and lower stiffness substrate [22]. Therefore, we used lentivirus-mediated expression of integrin β1 shRNA to validate whether higher matrix stiffness activates the GSK3β/β-catenin signaling pathway via integrin β1 and further influences OPN expression. Results showed that P-GSK3β and β-catenin levels were attenuated in HCC cells infected with LV-INTGB1-RNAi on high stiffness substrate. Nuclear expressions of β-catenin and TCF also dropped sharply, resulting in significant downregulation of OPN (Fig 2). All the above data demonstrated that matrix stiffness signal is transduced into HCC cells via integrin β1 and activates the GSK-3β/β-catenin pathway to upregulate OPN expression.

### GSK-3β inhibitor rescues the OPN expression in the infected HCC cells with LV-INTGB1-RNAi on high stiffness substrate

**Fig 3:**
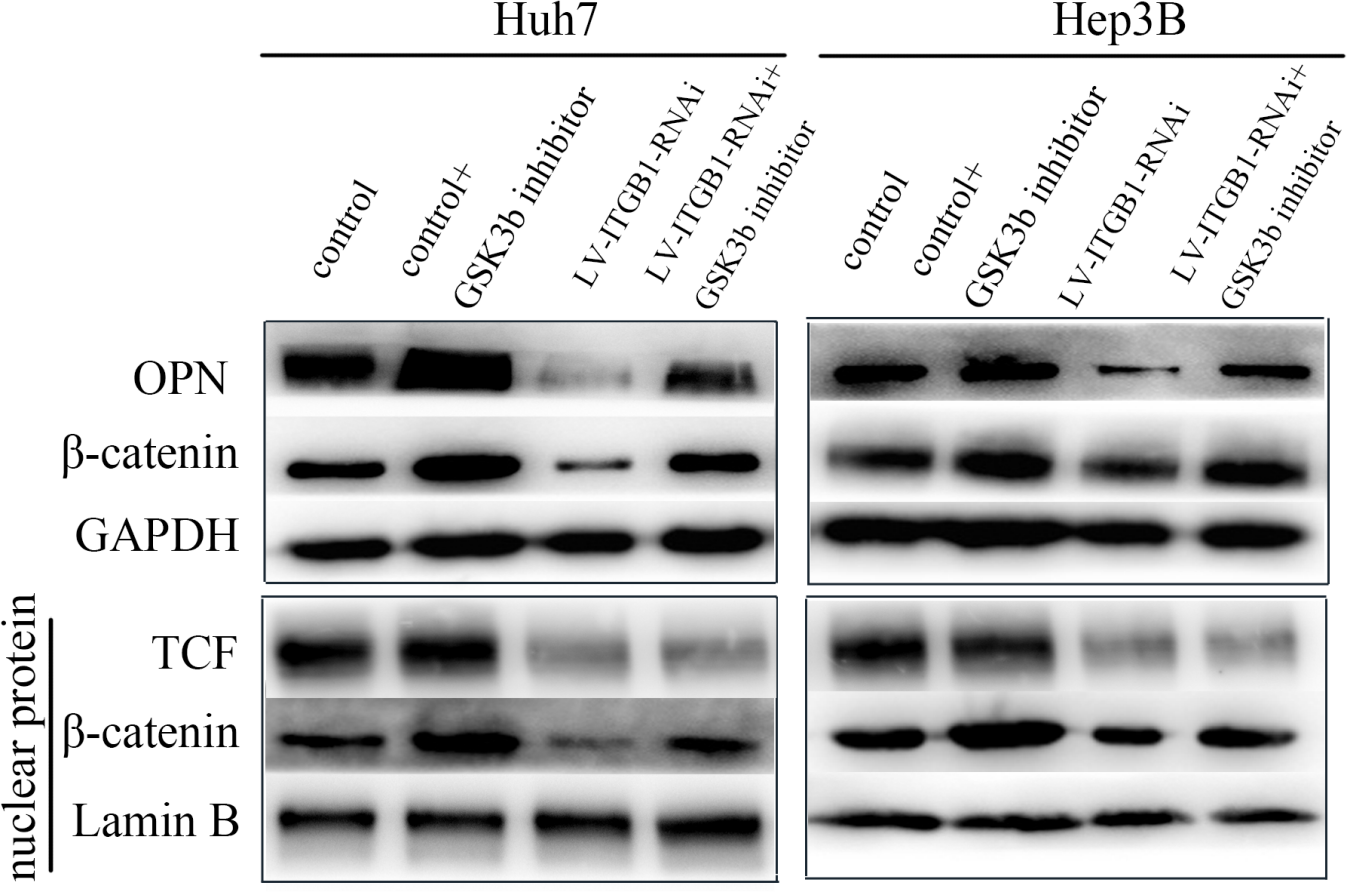
GSK-3β inhibitor rescues OPN expression in HCC cells infected with LV-INTGB1- RNAi on high stiffness substrate. In the infected HCC cells infected with LV-INTGB1-RNAi grown on high stiffness substrate, inhibition of GSK-3β reversed the previously described increase in OPN expression and β-catenin expression compared to the control cells.

The GSK3β inhibitor CHIR 99021 is a small organic molecule that can inhibit GSK3β by competing for its ATP-binding sites and can mimic the canonical β-catenin signaling pathway [31]. Knock-down of integrin β1 suppressed OPN expression and attenuated GSK3β/β-catenin pathway activation, as shown in Fig 2. The GSK3β inhibitor was further used to treat the infected HCC cells with LV-INTGB1-RNAi on high stiffness substrate for 48 h. The expression of OPN and β-catenin returned to levels similar to those untreated HCC cells. Meanwhile, expression of β-catenin and TCF in the nucleus was rescued in HCC cells (Fig 3). Additionally, compared with untreated cells, HCC cells treated with the GSK-3β inhibitor only expressed high levels of OPN and nuclear β-catenin expression, revealing a role for GSK3β inhibition in regulating OPN expression. Taken together, these results indicate that higher matrix stiffness can activate the integrin β1/GSK3β-β-catenin signaling pathway in HCC cells and subsequently upregulate OPN expression.

### Expression levels of OPN, LOX, and COL1 in HCC tissue with different matrix stiffness backgrounds

**Fig 4:**
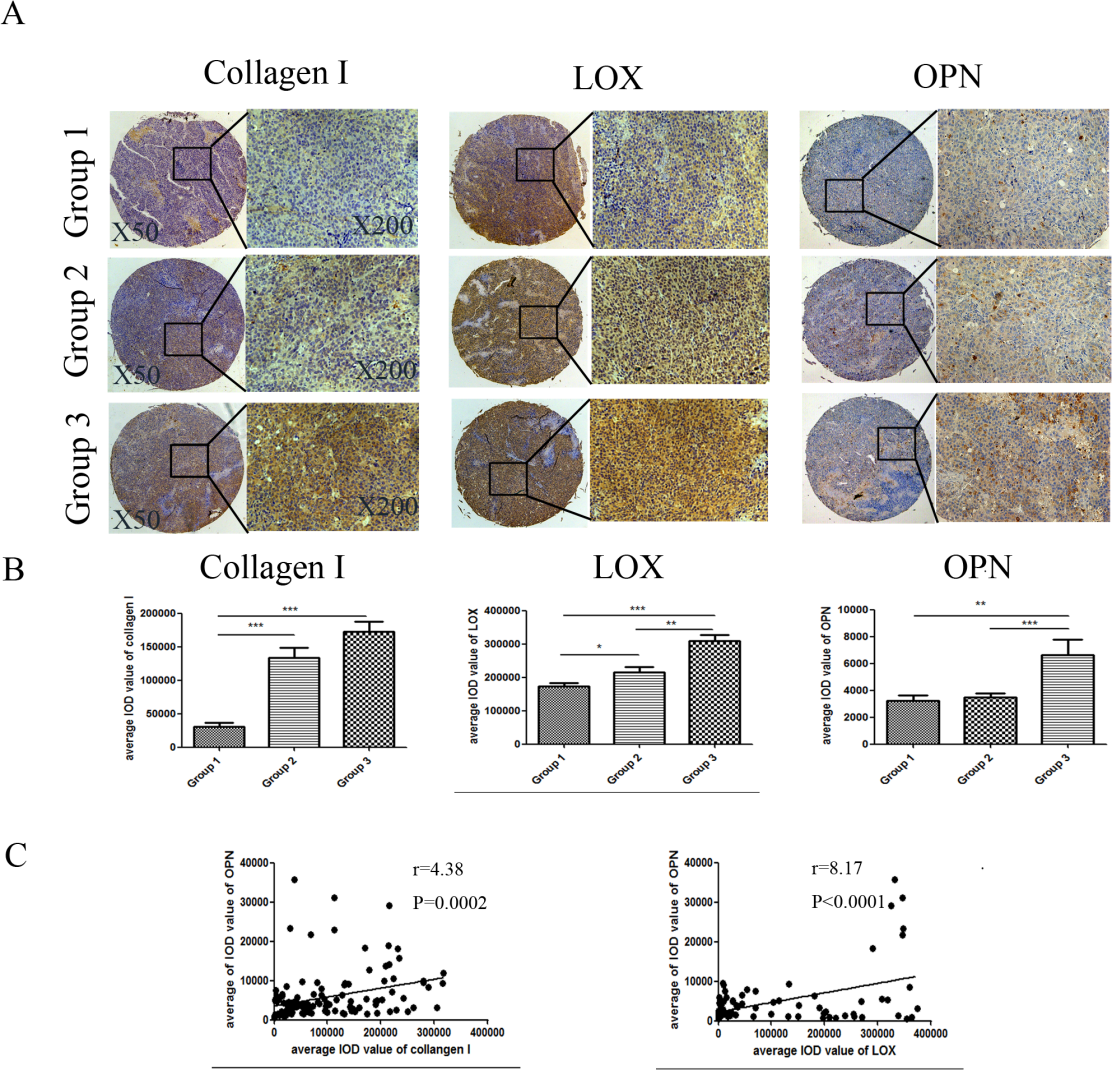
Expressions of COL1, LOX, and OPN in HCC tissue with different matrix stiffness backgrounds. (A) Representative HCC tumor samples show the expression levels of COL1, LOX and OPN. HCC tissue in Groups 1, 2, and 3 are defined as normal liver stiffness background tissue, medium stiffness and high stiffness liver background tissue, respectively[22]. (B) Graphs showing the average IOD value of COL1, LOX and OPN expression in the three groups analyzed by image pro-plus 6.0 software. In each case, error bars represent SD, *p < 0.05, **p < 0.01, ***p<0.0001. (C) Multiple linear regression indicates that the levels of OPN are positively correlated with the expression levels of LOX and COL1.

An HCC tissue microarray containing three groups of rat HCC models with different matrix stiffness backgrounds [22] was previously constructed to investigate the correlation between matrix stiffness and OPN expression. The expression levels of COL1 and LOX, which are commonly considered to be ECM stiffness indicators, were evidently different among the three groups. Moreover, their expression levels in HCC tissues with high and median stiffness backgrounds were remarkably higher than those of HCC tissues with low stiffness background (Fig 4A and 4B). In addition, OPN expression was also significantly increased in HCC tissues with higher matrix stiffness background. Multiple linear regression analysis showed that the expression levels of OPN were positively correlated with those of COL1 (r = 4.38) and LOX (r = 8.17). These tissue-level data further confirmed that increasing matrix stiffness facilitates upregulation of OPN.

## Discussion

Tumor microenvironment consists of stroma cells, cytokines, chemokines, proteinase, and extracellular matrix, all of which contribute to tumor initiation and progression [32–36]. Biochemical signals from extracellular matrix proteins, including collagen, fibronectin, and laminin, have well documented to be involved in HCC metastasis [37–39]. However, little is known about the underlying mechanisms of matrix stiffness modulated HCC progression. Based on various experimental culture systems with tunable stiffness [40–41], matrix rigidity were found to regulate chemoresistance [42], cell growth and dedifferentiation [43], tumor proliferation [44], invasion and metastasis [44–45], and angiogenesis [22]. Most studies to date mainly present the phenomenon or target other solid tumors, thereby the mechanism behind regulation of HCC invasion and metastasis by matrix stiffness remains unclear.

The level of plasma OPN significantly increases with advanced Child–Pugh class, large tumor size, high tumor grade, and late stage of HCC [46–48]. OPN overexpression is closely correlated with intrahepatic metastasis [49], early recurrence [50], and a worse prognosis [51], and is regarded as a potential prognostic biomarker for this disease. Downregulation of OPN suppresses growth of HCC via inhibiting apoptosis [52]. OPN is an attractive tumor marker for HCC because of its characteristics as an immobilized extracellular matrix molecule that is also present in secreted form in body fluids, including plasma and serum [53,54].

OPN can be regulated by Ras-activated enhancer, which binds to the T cell factor-4 binding site to promote OPN transcriptional activity [54,55]. β-catenin/Lef-1, Ets, and AP-1 transcription factors can stimulate transcription of OPN together in rat mammary cells [56]. In general, β-catenin/Tcf-Lef complex is considered as a transcription factor that activates OPN transcription. The interaction between hepatocyte growth factor and c-Met also increases OPN expression in human osteoblasts via the PI3K/Akt/c-Src/c-Jun and AP-1 signaling pathways [57]. However, hardly any studies have been conducted on matrix stiffness-mediated OPN expression. This study reveals that higher matrix stiffness can upregulate OPN expression via Wnt-independent-β-catenin pathway and offers a new insights on OPN regulation in HCC cells induced by physical mechanical signal. To our best knowledge, this study is the first to elucidate the mechanism underlying matrix stiffness-mediated effects on the regulation of OPN expression in HCC cells.

Using an in vitro system of mechanically tunable COL1-coated polyacrylamide gel, we initially investigated whether matrix stiffness regulates OPN expression in HCC cells. OPN expression was significantly upregulated in HCC cells at mRNA and protein levels with increasing matrix stiffness (Fig 1B). In addition, phosphorylation level of GSK3β and expression of nuclear β-catenin were also upregulated. This result indicates that Wnt-independent GSK3β/β-catenin pathway may be activated and involved in OPN regulation. Other studies have suggested that GSK-3β level plays a key role in controlling the amount of β-catenin in the cytoplasm [58]. Phosphorylation of GSK-3β participates in stabilizing β-catenin, which is a transcription coactivator for OPN expression [59]. Nuclear translocation of β-catenin can form a complex with TCF and bind to the promoter of OPN, thereby enhancing the translation of OPN. TCF/LEF transcription factors are the major end point mediators of Wnt/β-catenin signaling in mammals [60]. Transcription of TCF/LEF gene can be regulated by the Wnt pathway, and both genes are often identified as Wnt-regulated transcription factors in microarray studies [61]. In this study, matrix stiffness signal was found to activate GSK3β phosphorylation and result in nuclear translocation of β-catenin, which further increased OPN expression via the integrin β1 receptor. In addition, OPN expression and p-GSK3β level were all decreased in HCC cells with knock-down of integrin β1 grown on high stiffness substrate compared with that in the control cells. GSK-3β inhibition restrained β-catenin degradation, resulting in accumulation of β-catenin in the cytoplasm and even nuclear translocation (Fig 3). Moreover, OPN expression was also rescued in HCC cells infected with LV-ITGB1-RNAi. Taken together, higher matrix stiffness can activate integrin β1/GSK3β/β-catenin signaling pathway in HCC cells and upregulate their OPN expression.

**Fig 5:**
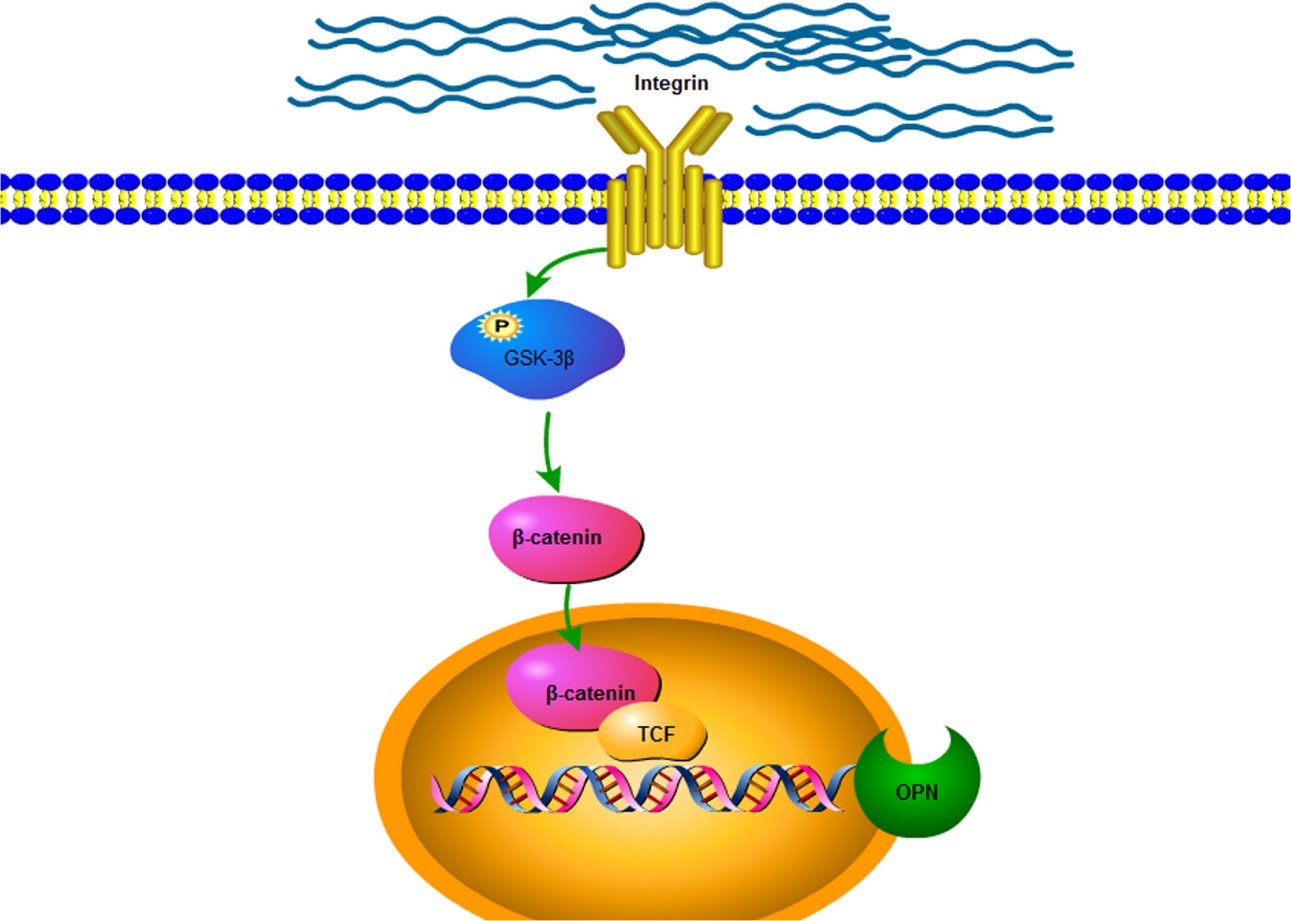
Schematic of the proposed mechanism by which matrix stiffness triggers the integrinβ1/GSK3β/β-catenin pathway to modulate OPN expression.

In this study, we present results that reveal a new Wnt-independent mechanism for regulating OPN expression in response to matrix stiffness (Fig 5). Other signaling pathways that co-regulate OPN expression along with the integrin β1/GSK3β/β-catenin signaling pathway may also exist. More studies are necessary to identify and elucidate them in the future.

## Conclusions

Our results suggest that higher matrix stiffness upregulats OPN expression in HCC cells mediated via the integrin β1/GSK3β/β-catenin signaling pathway.

## Supporting Information

S1 Fig Expression levels of invasion associated gene MMP9 under exogenous OPN intervention in HCC cells grown on different stiffness substrates. (DOCX)

S1 File Materials and Methods. (DOCX)
