# Supplementary material for: Higher Matrix Stiffness Upregulates Osteopontin Expression in Hepatocellular Carcinoma Cells Mediated by Integrin β1/GSK3β/β-Catenin Signaling Pathway
Source: PLoS One. 2015 Aug 17;10(8):e0134243. doi: 10.1371/journal.pone.0134243 (PMC4539226; doi:10.1371/journal.pone.0134243)
Supplement: S1 Fig — (DOCX) [file pone.0134243.s001.docx]

**Supporting Information**

**S1 Fig. Expression levels of invasion associated gene MMP9 under exogenous OPN intervention in HCC cells grown on different stiffness substrates**

Compared with the controls, exogenous human OPN upregulated MMP9 gene expressions of Huh7 cells cultured on different stiffness substrates. In each case, error bars represent SD, *p < 0.05 , **p < 0.01, ***p<0.0001.

**
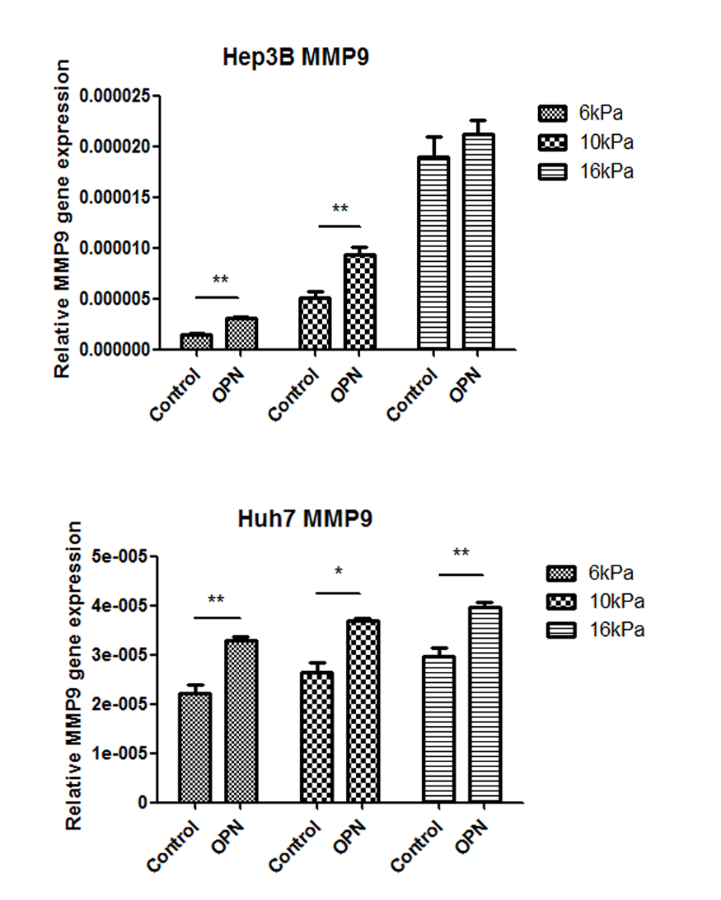
**
