# Supplementary material for: Higher Matrix Stiffness Upregulates Osteopontin Expression in Hepatocellular Carcinoma Cells Mediated by Integrin β1/GSK3β/β-Catenin Signaling Pathway
Source: PLoS One. 2015 Aug 17;10(8):e0134243. doi: 10.1371/journal.pone.0134243 (PMC4539226; doi:10.1371/journal.pone.0134243)
Supplement: S1 File — (DOCX) [file pone.0134243.s002.docx]

**Supporting Information**

**S1 File. Materials and Methods**

**Western blot**

After 48 h culture on COL1-coated polyacrylamide gels with tunable stiffness, HCC cells were collected using a cell scraper from the gel. The total proteins of HCC cells were extracted in the mixed lysis buffer containing RIPA buffer (Beyotime, China), 1 mM phenylmethanesulfonyl fluoride (Beyotime, China), and 10% PhosSTOP (Roche, Switzerland). Approximately 50 mg of total protein was loaded and separated by 10% SDS–PAGE and then transferred onto a polyvinylidene difluoride membrane (Millipore, USA). Subsequently, the membrane was blocked with 5% fat-free milk in TBS/Tween for 1 h at room temperature and reacted overnight at 4 °C with the following diluted primary antibodies: GSK3β (1:1000, Cell Signal Technology, Danvers, MA), p-GSK3β(1:1000, Proteintech, Wuhan, China), integrinβ1(1:1000, Cell Signal Technology, Danvers, MA), TCF (1:1000, Cell Signal Technology, Danvers, MA), β-catenin (1:1000, Cell Signal Technology, Danvers, MA), and OPN (1:500, Santa Cruz, USA), GAPDH (1:1000, Cell Signal Technology, Danvers, MA), Lanin B(1:1000, Cell Signal Technology, Danvers, MA). The membrane was further incubated with HRP-conjugated secondary antibody (1:1000, Dingguo Bio Beijing, China) for 1 h at room temperature. Finally, the target band was visualized using an electrochemiluminescence kit (Thermo, USA).

**Immunohistochemistry**

Immunohistochemical staining was performed as described in our previous work [2]. In a typical procedure, after rehydration and antigen retrieval, cell slides were incubated with diluted primary antibodies against OPN (1:50, Boster, China), LOX (1:100, Abcam, UK), COL1 (1:100, Abcam, UK) at 4 °C overnight, followed by horseradish peroxidase (HRP)-conjugated secondary antibody (antirabbit, 1:200; DingguoBio, Beijing) at 37 °C for 30 min. Finally, the slides were stained with 3,3’-diaminobenzidine and counterstained with Mayer’s hematoxylin. Staining intensity and the percentage of immunoreactive tissues were scored by two independent observers.

Photographs of four representative sites were captured under high-power magniﬁcation (×200) by the Leica QWin Plus v3 software with identical setting parameters. The density of positive staining was measured by Image-Pro Plus v6.2 software (Media Cybernetics Inc., USA). For the reading of each antibody staining, a uniform setting was applied to all slides. Integrated optical density of all the positive staining in each photograph was measured, and its ratio to the total area of each photograph was calculated.

**RNA extraction and real-time PCR assays for miRNA and mRNA detection.**

Total RNA was extracted from cultured cells with TRIzol Reagent (Invitrogen). The quality of RNA was examined by A260 absorption. For mRNA detection, 2 µg of total RNA were used for complementary DNA synthesis with a RevertAid/First Strand cDNA Synthesis Kit (Thermo scientific, Inc.). Real-time PCR was performed in triplicate using Platinum SYBR Green qPCR SuperMix-UDG (Invitrogen, Inc.). The primers for the genes of OPN (SPP1) were synthesized by Sangon Biotech Co., Ltd., as follows: 5’- CTCCATTGACTCGAACGACTC-3’(forward) and 5’- CAGGTCTGCGAAACTTCTTAGAT-3’(reverse).GAPDH: 5’-TGTGGGCATCAATGGATTTGG-3’ (forward) and 5’- ACACCATGTATTCCGGGTCAAT-3’ (reverse).

**Expression levels of invasion associated gene MMP9 under exogenous OPN intervention in HCC cells grown on different stiffness substrates**

Huh7 cells were cultured on 6kPa, 10kPa and 16kPa stiffness substrates and treated with recombinant human osteopontin (sino, biological, Inc.) with concentration of 0.6μg/ml 48h. Cells were collected for further real-time PCR assays of MMP9 genes.

**References**

1. Pelham. RJ. Jr, Wang. YI. Cell locomotion and focal adhesions are regulated by substrate flexibility. Proc Natl Acad Sci U S A. 94 (1997) 13661–13665.
2. Wang. YH, Dong. YY, Wang. WM, et al., Vascular endothelial cells facilitated HCC invasion and metastasis through the Akt and NF-κB pathways induced by paracrine cytokines. J Exp Clin Cancer Res. 32 (2013) 51.
